# Is preoperative pulmonary rehabilitation effective in the postoperative period after lung resection?

**DOI:** 10.4314/ahs.v23i1.69

**Published:** 2023-03

**Authors:** Hatice Kökez, Hakan Keskin, Makbule Ergin, Abdullah Erdoğan

**Affiliations:** Department of Thoracic Surgery, faculty of Medicine, Akdeniz university, Antalya, Turkey

**Keywords:** Thoracic, pulmonary rehabilitation, breathing exercise

## Abstract

**Objective:**

Investigating the effects of the preoperative short term intensive pulmonary rehabilitation program applied for patients who have undergone lung resection by thoracotomy, on lung functions, complication rates and length of hospital stay during the postoperative period.

**Methods:**

A prospective randomized trial of sixty patients were enrolled who would undergo pulmonary resection by thoracotomy and were randomly divided in two groups. Intensive pulmonary rehabilitation was performed on these patients in the study group 3 hours a day throughout 7 days during the preoperative period. Groups were compared with respect to their spirometric pulmonary functions, respiratory parameters, blood gas parameters, complication rates and length of hospital stay.

**Results:**

Total incidence rate of complications in the patients from the control group significantly increased(p=0,028). When patients who underwent lobectomy and wedge resection were observed, length of hospital stay of those in the control group was seen to be statistically higher in comparison with the study group(p<0,05).

**Conclusion:**

We consider that it will be very beneficial to perform a short term and intensive pulmonary rehabilitation program on every patient possible who is planned to undergo thoracotomy and lobectomy or wedge resection treatment.

## Introduction

The sole treatment method offering patients with lung cancer the longest survival time and the most effective therapy is, without any dispute, surgical therapy 1. Thoracotomy; is one of the surgical incisions with the highest pain complaint in the postoperative period due to anatomic structure of the thorax, intensive intercostal nerve sensation and its distribution. Shallow breathing, decreasing motion of thorax sourcing from post thoracotomy pain restricts ventilation in lungs and causes some, primarily pulmonary complications 2. In addition to these, the current respiration capacity will decrease even more during postoperative period due to the resected lung field^3^.

Anesthetic medicines applied and positive pressure ventilation generated by using endotracheal tube during surgery cause disruption of the tracheobronchial ciliary activity. Disrupted tracheobronchial ciliary activity causes retention of sputum and resulting atelectasis, having decreased required mucus transport towards the trachea^4^. Additionally, besides anesthesia, immobilization of the patient and staying in the same position throughout surgery, pain and diaphragm function anomaly decrease functional residual capacity (FRC) and promote airway collapse.

In patients who will undergo a pulmonary resection by thoracotomy; age, smoking history, history of cardiopulmonary diseases, weak pulmonary function test, low arterial blood gas results and low exercise capacity increase postoperative pulmonary complication risks^5^. These complications occurring during the postoperative period increase patient's length of stay in intensive care unit and hospital as well as their health expenses 6–9.

Pulmonary rehabilitation (PR) is a beneficial and necessary method to be applied, which enables patients to be actively involved in life in the preoperative and postoperative period, simplifying implementation of health services during subsequent processes and enabling patients to survive longer^10^. This way, the patient's tolerance to surgery and secretion drainage ability increase, diaphragmatic function develops and respiration need decreases^11^. All these provide a decrease in complication risks and symptoms and reduce the use of healthcare units which will improve life quality, physical and social activities^12^. In many controlled studies carried out, pulmonary rehabilitation practices have been proven to decrease length of hospital stay resulting in reduction of health expenses up to 35% for patients who have undergone pulmonary resection^13^,^14^.

We have planned to implement a prospective, randomized and controlled study with the aim of investigating the effects of preoperative short term and intensive pulmonary rehabilitation program performed on patients who have undergone pulmonary resection by thoracotomy, on pulmonary functions, complication rates and length of hospital stay during the postoperative period.

## Material and Methods

A prospective randomized trial of 60 patients who underwent pulmonary resection at Akdeniz University Hospital Thoracic Surgery Clinic between September-2016 and July-2017, were enrolled in the study. Average age of the patients was 54,8(18-65 age), 30 out of which were women and 30 of which were men. All patients enrolled were given detailed information about the study and a signed confirmation form was received from each patient. An approval was received from ‘Akdeniz University, Faculty of Medicine, Ethics Committee for Clinical Studies’ in order to carry out the study.

Age, gender, body mass index (BMI) of the patients, their length of hospital stays, type of the operation made and postoperative complications such as atelectasis, prolonged air leak and arrhythmia were recorded.

The patients were divided into 2 groups as control group and study group and each group consisted of randomly selected 30 patients. 30 patients in the study group were given respiration exercise training, intensive spirometer training and applied bi-level positive airway pressure(BIPAP) 3 hours a day for 7 days, while the other 30 patients in the control group were not provided with any training on preoperative pulmonary rehabilitation.

In the postoperative period, after the patients were extubated, both groups were applied the same pulmonary rehabilitation methods which are routine practices. Patients did respiration exercises and applied incentive spirometer for 4-6 hours a day while in intensive care and 2-4 sessions a day during their stay in the hospital ward. BIPAP was performed as in 3 sessions for 3 days after the operation. In the preoperative period, hemodynamic parameters were measured, respiratory function test (RFT) and arterial blood gas (ABG) test were made for all the patients. paCO2 and paO2 values were studied during ABG test and FEV1 and FVC values were studied during RFT test. Effectiveness of the study was investigated by carrying out measurements by RFT and ABG, at the 4th, 12th, 24th and 48th hours following extubation in the postoperative period. In the postoperative period, the patients who showed severe hemodynamic disturbance and psychiatric problems to the extent of avoiding their participation in the study were excluded.

In the preoperative period, patients in the study group were asked to apply the following respiratory exercise plan;

With the aim of training and providing their adaptation to the study, the patients were given information about the operation to be made, the region and position to be operated, postoperative pain, endotracheal tube, drainage tubes, oxygen therapy and intensive care process.

Information was provided about effectiveness of breathing exercises, incentive spirometer and BIPAP application in providing sufficient amount of ventilation and secretion drainage, due to the fact that the operation to be made as well as the anesthesia and mechanical ventilation during the operation will decrease the respiration capacity. For this purpose, the following exercises were done by the patients for 7 days as 3 hours a day.

1. Breathing exercises

a. Diaphragmatic breathing exercises 10 repeat/day

b. Segmental breathing exercises (unilateral, posterior, bilateral basal and apical) 10 repeat/day

c. Puckered lip breathing 10 repeat/day

2. Use of incentive spirometer 15 repeat/day

3. Coughing technique

4. BIPAP application 20 min/day

In statistical analysis SPSS (Statistical Package for Social Science Version 20) was used and distribution according to age, gender, BMI as well as smoking history of the groups were studied with ‘student t’ test, ABG and RFT parameters were studied with Repeated Measures ANOVA, student t’ test, their length of stay in hospital was studied with the comparative ‘Mann Whitney U Test’ while the complication rates were studied with ‘Fisher's Exact Test, Pearson chi-square test’ and p <0,05 was considered to be statistically significant.

## Results

When demographic data about these 60 patients were compared as control and study group, there was no significant difference observed. Average age of the patients was 54,8±8,72 in the control group and 54,8±11,18 in the study group. While the control group consisted of 10 women (33,3%), and 20 men (66,7%), the study group consisted of 8 women (26.7%) and 22(73,3%) men. Number of patients with a value of BMI&g;25 kg/m2 was 19(63,3%) in the control group, while it was 18(60%) in the study group.

**Figure 1:**
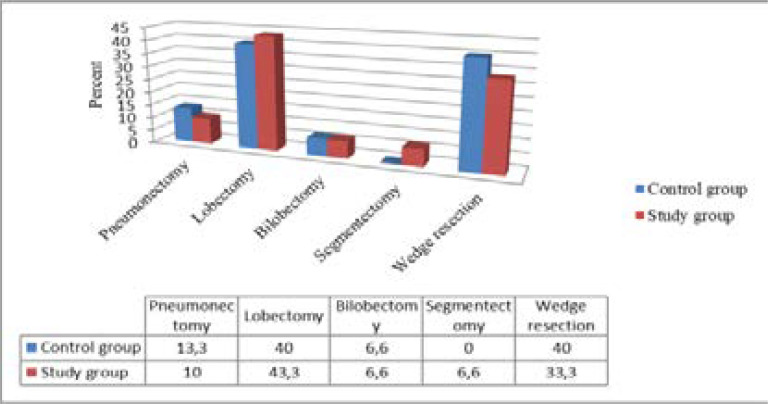
Distribution of pulmonary resection types

Types of pulmonary resection in the control and study group were compared in terms of their distributions and the obtained findings were presented. While 4 pneumonectomy, 12 lobectomy, 12 bilobectomy, 12 wedge resection procedures were performed in the control group, 3 pneumonectomy, 13 lobectomy, 2 bilobectomy, 2 segmentectomy and 12 wedge resection procedures were performed in the study group. There was no difference detected in pulmonary resection variety between the groups (p>0,05). Distribution of pulmonary resection types across the groups are given in figure 1.

Table 1 displays the findings obtained through comparison of the preoperative pCO2, pO2, FEV1 and FVC parameters of the control and study groups. And the table 2 displays postoperative pCO2 and pO2 values of the control and study groups. Based on the findings, statistically no significant difference was found between preoperative and postoperative parameters of the control and study groups (p>0,05).

**Table 1:** Comparison of preoperative blood gas parameters and respiratory function tests according to the control and study group

	Control	Study	P
pCo_2_, median(min-max)	33,85(26,8–47,5)	34,9(29,1–51,4)	0,706
pO_2_, av±SD	89,68±13,11	85,87±14,33	0,287
fev1, av±SD	2,49±0,66	2,6±0,96	0,615
fvc, av±SD	2,86±0,74	3,16±1,01	0,208

**Table 2:** Comparison of postoperative blood gas parameters according to the control and study groups

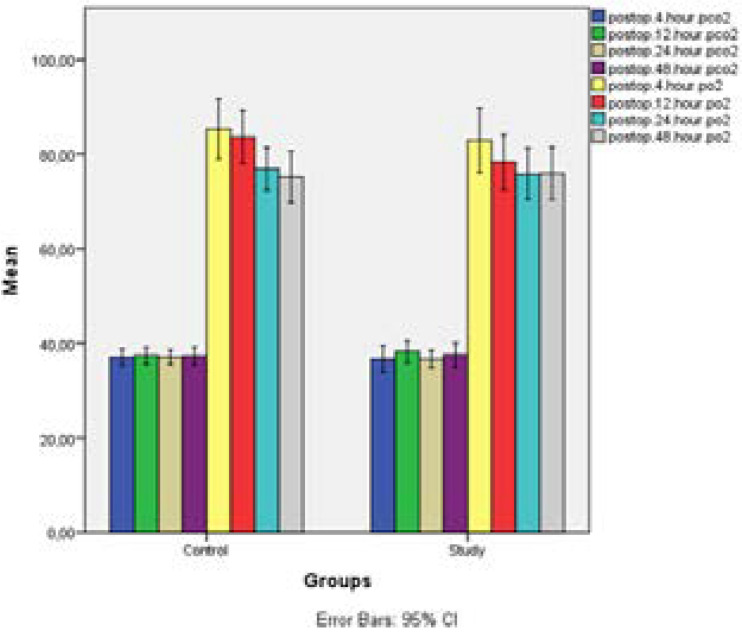

When changes in pCO2 ve pO2 values of the control and study group were analysed based on time periods, statistically changes neither in pCO2 (p=0,795) nor PO2 values showed any significant difference (p=0,680). When change in pCO2 value was evaluated separately by time in the control and study group, it was observed that statistically there was no significant change in any of the two groups (p>0,05). While statistically no significant difference was observed between pO2 values at the 4th hour and pO2 values at the 12^th^ hour in the control group, pO2 values at the 24^th^ and 48^th^ hours were observed to be lower than pO2 values at the 4^th^ hour (p=0,012). Similarly, while an increase was detected in pO2 value by time in also the study group, it was established that this change was statistically not significant (p=0.167). (Table 2)

Table 3 indicates changes in the parameters of postoperative respiratory function with time in the control and study groups. No significant difference was observed between changes in FEV1 (p=0,573) and FVC (p=0,147) values by time in the control and study groups. FEV1 values of the study group taken at the 4^th^, 12^th^ and 48^th^ hours were observed to be higher than those in the control group while statistically the difference was not significant (p>0,05). Similarly, there was no significant difference found between FVC values of the control and study groups taken at the 4^th^, 12^th^ and 48^th^ hours (p>0,05).

**Table 3:** Comparison of postoperative respiratory function test parameters of the control and the study groups

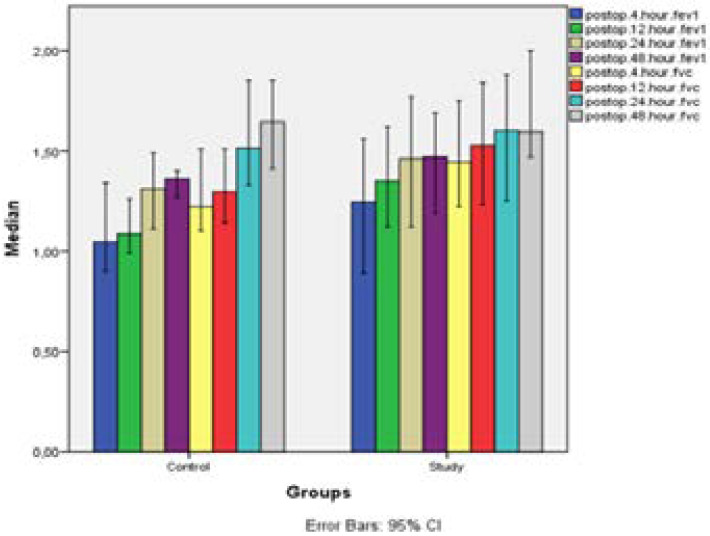

Postoperative complication rates of the study and control groups were compared as in table 4. No difference was observed between two groups in terms of atelectasis, pneumonia and arrhythmia complications (p>0,05). However, prolonged air leak was observed in 8 patients (26,7%) in the control group, while it was not observed in any of the patients in the study group. This difference was considered statistically significant (p=0,005). Therefore, total incidence rate of complications in patients from the control group significantly increased depending on this difference (p=0,028).

**Table 4:** Comparison of postoperative complication rates of the control and study groups (1Fisher's Exact test, 2Pearson chi-square test)

		Control n(%)	Study n(%)	P
Atelectasis	+	1(3,3)	1(3,3)	0,999^1^
	-	29(96,7)	29(96,7)	
Pneumonia	+	3(10)	2(6,7)	0,999^1^
	-	27(90)	28(93,3)	
Prolonged air leak	+	8(26,7)	0(0)	**0,005** ^1^
	-	22(73,3)	30(100)	
Arrhythmia	+	0(0)	1(3,3)	0,999^1^
	-	30(100)	29(96,7)	
Total Complication	+	10(33,3)	3(10)	**0,028** ^2^
	-	20(66,7)	27(90)	

In total 25 patients were applied lobectomy, 12 out of which were in the control group while 13 were in the study group. Prolonged air leak developed in only 8 patients in the control group and this result was found to be statistically significant (p=0,039). Also, postoperative complication rate of other pulmonary resection types was compared between the control and study groups. Statistically no significant difference was observed between the groups (p>0,05).

Table 5 displays the results related to the comparison made between the median length of hospital stay in different resection types across the control and study groups. According to this, it was observed that for patients who underwent lobectomy and wedge resection, length of hospital stay of those in the control group was statistically higher in comparison with those in the study group (p<0,05). While the median length of hospital stay of the control group was 10 days (4-24days), this period was found to be 6 days (3-21 days) for the study group which is statistically significant (p=0,001).

**Table 5:** Comparison of the median length of stay of the control and study groups on basis of resection types (*Mann Whitney U test (p<0,05))

		Control	Study	p*
Resection type	Pneumonectomy	14,5(10–24)	13(7–19)	0,724
	Lobectomy	10(6–17)	6(5–11)	0,004*
	Bilobectomy	17,5(11–24)	14(7–21)	0,439
	Segmentectomy	-	7,5(7–8)	-
	Wedge rezection	8(4–16)	5,5(3–20)	0,021*
Total		10(4–24)	6(3–21)	0,001*

When comorbidities were compared, 8 patients with hypertension, 3 with asthma, 2 with COPD had a total of 13 chronic diseases in the control group, 6 patients with hypertension, 2 with asthma, 1 with COPD had a total of 9 chronic diseases in the study group. No significant difference was found when the two groups were compared in terms of comorbidity.

## Discussion

Thoracic surgery contains patient groups requiring close follow-up and treatment after surgery, due to the respiratory functions adversely affected from incisions and operation types carried out. Besides adverse effects of pulmonary resection performed, high concentrated oxygen intake, single-lung ventilation, patient staying in the same position and being immobile throughout surgery, diaphragmatic dysfunction cause respiratory functions to severely decrease and air way collapse. Anesthetic agents disrupt mucociliary activity and this results in decrease of mucus transport avoiding secretion drainage through the bronchioles while triggering pulmonary complications such as atelectasia. Also, thoracotomy causes serious postoperative pain and avoids secretion drainage by disabling patients to cough effectively and by obstructing deep breathing 2,3,4.

Rate of pulmonary complications occurring during changes in respiration after pneumotomy considerably increases. And It has been established that these pulmonary complications increase the length of hospital stay, hospital expenses as well as morbidity and mortality rates 10. Various studies made have proven that breathing exercises are helpful to prevent these complications, bringing respiratory functions up to a sufficient level and for patients to continue with their active lives. Especially since 1960s, many studies have been made on benefits and necessity of pulmonary rehabilitation performed on patient groups who would undergo a surgery in the postoperative period^15^,^16^,^17^. However recently some opinions have started to be pronounced claiming that besides respiratory rehabilitation in the postoperative period, respiratory rehabilitation techniques carried out during the preoperative period are decreasing morbidity rates possible to occur during the postoperative period. 15,16,17

Based on this, 30 patients in the study group underwent pulmonary rehabilitation 3 hours a day throughout 7 days during the preoperative period. The program included breathing exercises, coughing technique exercise, incentive spirometry and BİPAP. While in some studies, PR was applied intensively for 1 week, in other studies it was applied for 4-6 weeks. And in our study, it was applied intensively for 1 week. In literature, it has been recorded that a 4–6-week PR would be difficult due to patient's unwillingness and since it delays the operation 18.

The study group consisting of 10 patients underwent intensive physiotherapy during preoperative period. In the study made by Pehlivan et al on 60 patients with non-small cell lung cancer where they investigated the effects of short-term intensive physiotherapy on pulmonary functions, intensive physiotherapy was performed on the patients in the study group consisting of 30 patients 19. No difference was detected between two groups in pulmonary function tests however, a severe difference was observed in oxygen saturation levels. No difference was observed with respect to complications however, a considerable decrease was stated in length of hospital stay. Unlike this, no difference was detected in RFT and ABG in our study however, there was difference detected in complication rates and length of hospital stay.

Postoperative complication rates and length of hospital stay were compared in two randomized studies carried out by Benzo et al. on patients who underwent lung cancer resection and who had a mid level, heavy COBD history. In the first study, a comparison was made between a 4-week preoperative conventional PR program and a standard maintenance therapy 18. However, in this study it was stated that there were some difficulties experienced about participation in the study because patients and health team were reluctant to postpone the operation. No significant difference was detected between the groups (n=9). And during the second study, the study group was offered self-sufficiency, inspiratory muscle training and slow breathing exercises and the control group (n=9) was offered a standard therapy. In the study group, a decrease was observed in the length of hospital stay and number of days with the need of chest tube. Two randomized studies made revealed that a short PR treatment have some potential while as stated it is not easy to implement a 4-week preoperative conventional PR program. We limited our preoperative study time with 1 week for simplicity of application and since we believe time should not be extended much for patients with tumor. While, as we can see, there was no significant result obtained as a result of the 4-week study made by Benzo et al, we observed a decrease in complication rates and length of hospital stay in our groups as a result of a 1-week intensive PR application.

In the randomized controlled study analysing effect of high intensity preoperative pulmonary rehabilitation treatment on old aged patients carried out by Licker et al, an intensive pulmonary rehabilitation was performed on the patients in study group (n=74) while the control group (n=77) underwent standard maintenance therapy^20^. At the end of the study a clear improvement was detected in the exercise capacity of the group who underwent an intensive PR however, it failed in reducing early complication incidence rate following lung cancer resection. In contradiction to this result, in the group containing 25 patients who underwent lobectomy, prolonged air leak was observed only in 8 patients who did not undergo preoperative PR and this difference was significant (p=0,039). According to this, we believe PR must certainly be applied prior to major surgeries especially such as lobectomy, where expansion failure can be higher and parenchyma loss can be more.

Saito et al. investigated the effect of preoperative pulmonary rehabilitation on postoperative complication incidence in patients with chronic obstructive respiratory disease (COPD) 21. Records of 116 patients with COPD were studied and retrospective records of 51 patients undergoing preoperative PR were studied. PR period was established as 18,7±12,7 days. In PR group; a significant improvement was observed in pulmonary functions (VC %5,3, FEV1 %5,5; P<0.05) and a decrease was detected in postoperative complication incidence rate (p<0.05) As a result of the research, it was stated that PR increases recovery speed of pulmonary functions in early stages and decreases postoperative complications.

In a study made by Sekine et al on COBD patients suffering from lung cancer who had undergone lobectomy, a two-week intensive physiotherapy was performed on the study group which continued throughout postoperative period 22. Although FEV1 and FEV1/FVC values were low for the study group in the preoperative period, these values did not increase in the postoperative period but remained and increase in postoperative pulmonary complication incidence rate and extension of the length of hospital were prevented. It is stated that although FEV1 and FEV1/FVC values were low for the study group in the preoperative period, these values did not decrease in the postoperative period but remained and increase in postoperative pulmonary complication incidence rate and extension of the length of hospital were prevented. And in our study, FEV1 values measured at the 24^th^ and 48th hours in the study group were higher than FEV1 values measured at the 4th hour, FEV values measured at the 48th hour was higher than the values measured at the 12th hour (p=0,005).

We believe that segmental breathing exercises, diaphragmatic breathing exercises, puckered lip breathing, incentive spirometer and BIPAP application within the scope of intensive pulmonary rehabilitation program heal pulmonary functions and reserves, increase exercise tolerance, decrease postoperative complication incidence and reduce the length of hospital stay. Additionally, we believe along with the training provided in the postoperative period, pain tolerance and adaptation to the rehabilitation process increase.

In the study made by Skinner EH on aged patients with lung cancer who have undergone surgery, effects of a 7-day preoperative pulmonary rehabilitation on pulmonary functions and length of hospital stay have been assessed. Aerobic endurance exercise, breathing exercises were applied for the study group (n=30) and a standard maintenance therapy was applied for the control group (n=30). At the end of the study, frequency of the postoperative complications was found to be lower and length of hospital stay was established to be shorter in the study group (average 3,8 days). In our study, a 7-day intensive rehabilitation was applied however, incentive spinometer and BIPAP application were performed instead of aerobic endurance exercises. As a result of the assessments, a significant decrease was recorded in the postoperative complication incidence rate and length of hospital stay.

In the literature reviews, there are studies indicating that preoperative pulmonary rehabilitation applications have a positive effect on pulmonary mechanisms 18,19,22,24. According to this data, rehabilitation program of many studies includes breathing exercises, inspiratory muscle exercises, aerobic endurance exercises, incentive spinometer, swimming exercises, coughing exercises. In our study, besides breathing exercises, application of BIPAP is performed as well together with these other methods. It was used to improve pulmonary functions and increase the reserves in the preoperative and postoperative periods. Our objective was to prevent development of postoperative pulmonary complications. Accordingly, in our study, FEV1 and FEVC values of the study group measured at the 4^th^, 12^th^, 24^th^ and 48^th^ hours were observed to be higher than those of the control group but it was determined that the difference was statistically not significant (p>0,05). Additionally, in the study group, non-incidence rate of complication was found to be lower within the preoperative complication rate (p=0.028).

## Limitation

This study has several limitations. It is a single center study and a limited number of cases were analysed. Evaluation of some rare complications could not be made clear because it was studied with a limited number of patients.

## Conclusion

BIPAP application within the scope of rehabilitation program can be applied for increasing lung mechanics in the preoperative period. The study reveals that besides rehabilitation applied in the postoperative period, it will affect the patients' therapy process in a positive way if applied in the preoperative period as well. We believe especially a 1-week short but intensive PR program is more effective in comparison with long term PR programs with regards to the patient and implementing team.

At the end of this study made in the patient group who underwent pulmonary resection by thoracotomy, we established that respiratory rehabilitation program applied in the preoperative period decreases prolonged air leak and consequently postoperative complication incidence especially in patients who have undergone lobectomy and at the same time, it shortens the length of hospital stay in patients who have undergone lobectomy wedge resection. We argue that it would be beneficial to apply a short term, intensive PR program for each patient who is planned to undergo a thoracotomy and especially a resection such as lobectomy and wedge.

## References

[1] Kaynak K (2007). Akciğer Kanserinde Cerrahi Tedavi. İ. Ü. Cerrahpaşa Tıp Fakültesi Sürekli Tıp Etkinlikleri.

[2] Günlüoğlu MZ (2010). Postoperatif Pulmoner Komplikasyonlar. Journal of Clinical and Analytical Medicine Kitap Serisi, Akciğer Hastalıkları ve Tedavisi.

[3] Bastin R, Moraine JJ, Bardocsky G (1997). Incentive spirometry performance. A reliable indicator of pulmonary function in the early postoperative period after lobectomy?. Chest.

[4] Swenson ER, Swenson ER, Albert RK, Spiro SG, Jett JR (2004). Ch 17 Preoperative pulmonary evaluation. Clinical Respiratory Medicine.

[5] Duggan M, Kavanagh BP (2010). Perioperative modifications of respiratory function. Best Pract Res Clin Anaesthesiol.

[6] Feltracco P, Serra E, Barbieri S (2012). Postoperative Care of Patients Undergoing Lung Resection. J Anesthe Clinic Res.

[7] Stéphan F, Boucheseiche S, Hollande J (2000). Pulmonary complications following lung resection: a comprehensive analysis of incidence and possible risk factors. Chest.

[8] Varela G, Ballesteros E, Jiménez MF (2006). Cost effectiveness analysis of prophylactic respiratory physiotherapy in pulmonary lobectomy. Eur J Cardiothorac Surg.

[9] Kılıçgün A, Gökçe M (2013). Ameliyat Sonrası Görülen Komplikasyonlar. Göğüs Cerrahisi. 2. Baskı.

[10] Özalevli S (2009). Pre ve Postoperatif Pulmoner Rehabilitasyon. In: Erk M, Ergun P; Eds. Pulmoner Rehabilitasyon. Toraks Kitapları.

[11] Takaoka ST (2005). The Value of Peroperatif Pulmonary Rehabilitation. Thorac Surg Clin.

[12] Mahler DA (1998). Pulmonary Rehabilitation. Chest.

[13] Varela G, Novoa NM, Agostini P (2011). Chest physiotherapy in lung resection patients: state of the art. Semin Thorac Cardiovasc Surg.

[14] Yüksel M (2001). Göğüs Cerrahisi.

[15] Ambrosino N, Gabbrielli L (2010). Physiotherapy in the perioperative period. Best Pract Res Clin Anaesthesiol.

[16] Reeve JC (2008). Physiotherapy interventions to prevent postoperative pulmonary complications following lung resection. What is the evidence? What is the practice?. New Zealand Journal of Physiotherapy.

[17] Shannon VR (2010). Role of pulmonary rehabilitation in the management of patients with lung cancer. Curr Opin Pulm Med.

[18] Benzo R, Wigle D, Novotny P (2011). Preoperative pulmonary rehabilitation before lung cancer resection: results from two randomized studies. Lung Cancer.

[19] Pehlivan E, Turna A, Gürses A (2011). The effects of preoperative short-term intense physical therapy in lung cancer patients: a randomized controlled trial. Ann Thorac Cardiovasc Surg.

[20] Licker M, Karenovics W, Diaper J (2017). Short-Term Preoperative High-IntensityInterval Training in PatientsAwaiting Lung CancerSurgery: A RandomizedControlled Trial. J Thorac Oncol.

[21] Saito H, Hatakeyama K, Konno H (2017). Impact of pulmonary rehabilitation on postoperative complications in patients with lung cancer and chronic obstructive pulmonary disease. Thorac Cancer.

[22] Sekine Y (2005). Perioperative Rehabilitation and Physiotherapy for Lung Cancer Patients with Chronic Obstructive Pulmonary Disease. The Japanese Journal of Thoracic and Cardiovascular Surgery.

[23] Skinner EH (2017). Intensive preoperative rehabilitation improves functional capacity and postoperative hospital length of stay in elderly patients with lung cancer. J Fizyot.

[24] Morano MT, Araújo AS, Nascimento FB (2013). Preoperative pulmonary rehabilitation versus chest physical therapy in patients undergoing lungcancer resection: a pilot randomized controlled trial. Arch Phys Med Rehabil.

